# Sonographic pediatric liver size standards based on generalized additive modeling in a diverse population

**DOI:** 10.1007/s00247-026-06612-3

**Published:** 2026-04-24

**Authors:** Amirreza Manteghinejad, Marcus Meneses, Julian Lopez Rippe, Erica L. Riedesel, Summer L. Kaplan

**Affiliations:** 1 Department of Radiology, Children’s Hospital of Philadelphia, 3401 Civic Center Boulevard, Philadelphia, PA 19104 USA; 2 University of Pennsylvania, Philadelphia, USA

**Keywords:** Child, Diagnostic Imaging, Liver, Organ Size, Reference Values, Ultrasonography

## Abstract

**Background:**

In pediatric radiology, assessment of liver size is a fundamental component of abdominal imaging, reflecting both normal hepatic growth and potential pathologic processes. Limitations in prior literature, along with evolving demographic characteristics of the pediatric population, highlight the need for updated, methodologically rigorous reference ranges for normal liver size on ultrasound.

**Objective:**

This study aims to establish contemporary ultrasound-based reference ranges for pediatric liver size, incorporating both age and height as key variables.

**Materials and methods:**

In this retrospective study, radiology reports from January 2014 to December 2024 for patients under 18 years of age were reviewed. Patients with an impression of “normal abdominal ultrasound” were included, excluding those with liver diseases or abnormal Aspartate Aminotransferase (AST) and Alanine Aminotransferase (ALT) results. Standard liver length measurement for our institution is the craniocaudal dimension of the right lobe along the midclavicular line. Generalized Additive Models for Location, Scale, and Shape (GAMLSS) with the Box–Cox Power Exponential (BCPE) distribution was used to create reference ranges based on patients' age and height.

**Results:**

A total of 4,611 abdominal ultrasound examinations met the inclusion criteria. The study included 2,055 males (44.6%) and 2,556 females (55.4%). At birth, the liver size ranged from 4.9 to 5.5 cm (cm), increasing progressively with age to a maximum of 17.2 cm in adolescents aged 17–18 years. A subset of 3,235 examinations (70%) with recorded height was used for GAMLSS modeling, showing a more linear increase in liver size with height. Reference ranges were reported up to 180 cm in height, with a maximum liver size of 17.5 cm.

**Conclusion:**

This study provides updated reference ranges and nomograms for pediatric liver size based on age and height, which support a normal liver size slightly higher than previously reported.

**Graphical Abstract:**

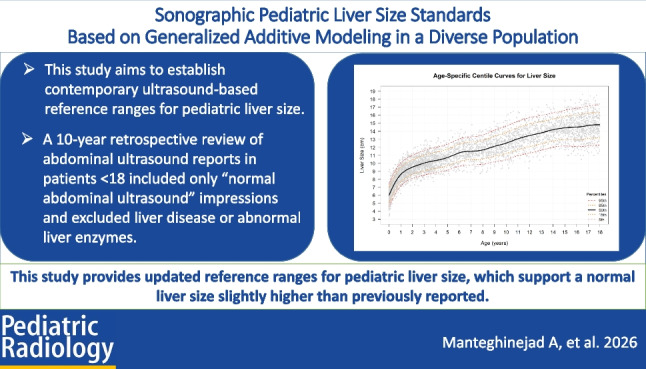

**Supplementary Information:**

The online version contains supplementary material available at 10.1007/s00247-026-06612-3.

## Introduction

In pediatric radiology, assessment of liver size is a fundamental component of abdominal imaging as it reflects both normal growth of the liver and potential pathologic processes [1, 2]. Abnormal liver size may be the earliest or only imaging sign of a broad spectrum of hepatic diseases, including metabolic, infectious, vascular, and infiltrative disorders [3]. Physical examination provides only a rough estimate of liver size in children and is limited by several factors. In infants and young children, the liver edge is often normally palpable up to 2 cm (cm) below the right costal margin, making clinical detection of mild hepatomegaly unreliable [4]. The assessment is also highly operator-dependent; even among trained clinicians, inter-observer variability in estimating the liver edge is substantial, and the true upper and posterior margins of the liver cannot be defined by palpation alone [5]. Body habitus, respiratory motion, and patient cooperation further limit accuracy. Moreover, subtle or segmental enlargement and early parenchymal disease may not be apparent on examination and broader studies of abdominal examination findings in children demonstrate generally poor inter-examiner reliability [1].

Diagnostic imaging, particularly ultrasound, is the preferred method for objective and reproducible assessment of liver size in children due to its safety, accessibility, and reproducibility [1, 6]. However, interpretation of liver size is dependent on population-appropriate reference ranges. This is challenging in children as liver dimensions change dynamically with age and are influenced by age, height, weight, body surface area, sex, and ethnicity [7].

A recent systematic review summarized published data up to 2020 on normal pediatric liver, spleen, and kidney size reported on ultrasound [1]. This review highlighted substantial heterogeneity in study methodologies, measurement techniques, population characteristics, and even discrepancies in measurements for the same age group between some of the studies. Many studies were limited by small sample sizes and inconsistent adjustment for anthropometric variables [1].

Given the limitations of prior literature and the evolving demographic characteristics of the pediatric population [1], there is a clear need for updated, methodologically rigorous reference ranges for normal liver size on ultrasound in children. This study aims to create an updated ultrasound normal reference range of liver size in the pediatric population, accounting for age and height as variables.

## Materials and methods

A retrospective cross-sectional study was performed at a quaternary-care pediatric hospital. The institutional review board of the hospital approved the study, and the need for consent forms was waived due to the retrospective nature of this study.

Radiology reports in the hospital’s electronic health record over a 10-year period (January 2014 through December 2024) were queried to identify patients < 18 years of age who underwent complete abdominal ultrasound. Only studies with an impression of “normal abdominal ultrasound” were included. Reports mentioning any space-occupying lesion, abnormal echotexture, or other pathological findings were excluded. Right upper quadrant ultrasound exams were also excluded, as these are more likely to be performed for suspected or known hepatobiliary pathology.

After identifying patients with the impression of normal abdominal ultrasound, any patients with documented liver diseases, infectious hepatitis, malignancy of liver or intrahepatic bile ducts, genetic and metabolic liver diseases, and conditions known to affect hepatic size (e.g., heart failure) were also excluded. The full list of excluded diagnoses and corresponding International Classification of Diseases (ICD-10) codes is provided in Online Resource 1.

Laboratory records of the patients were also reviewed; Patients with a documented abnormal aspartate aminotransferase (AST) or alanine aminotransferase (ALT) result were also excluded, regardless of the lab test date.

If a patient had more than one eligible ultrasound examination, all qualifying studies were included in the analysis.

Illuminate Insight (Softek Illuminate; Kansas City, KS) software was utilized to retrieve radiology reports and ultrasound data. Patient height and other demographics, including race and ethnicity, were obtained from the Epic (Epic Systems Corporation, Verona, Wisconsin, USA) electronic health records. Heights were included only when the measurement occurred within the appropriate age-specific interval relative to the ultrasound date: < 2 years, ± 90 days; 2– < 9 years, ± 180 days; 9– < 14 years, ± 90 days; and ≥ 14 years, ± 180 days. When multiple heights were available within this timeframe, the value closest to the ultrasound date was used.

Liver size values were parsed from free-text reports using regular expressions (Regex) and standardized to centimeters.

Outlier detection was done in a two-step process. First, any measurements smaller than 3 cm or larger than 25 cm were excluded as outliers. Second, two distribution-agnostic rules were utilized to flag recorded liver sizes: (1) z-scores based on the median and median absolute deviation (MAD-z), with |z_r_|≥ 3.5 considered extreme and 3 ≤|z_r_|< 3.5 considered moderately unusual [8]; and (2) Tukey fences, with values outside 1.5 × interquartile range (IQR) flagged as moderate and outside 3 × IQR as extreme [9]. For both rules, age-stratified and height-stratified groups were used. For age stratification, year 1 was divided into four 3-month bins, year 2 into two 6-month bins, and subsequently into annual bins. For height stratification, the bins were defined in increments of 10 cm. Measurements meeting any extreme threshold or two moderate thresholds underwent manual review by a radiology research scholar.

**Fig. 1:**
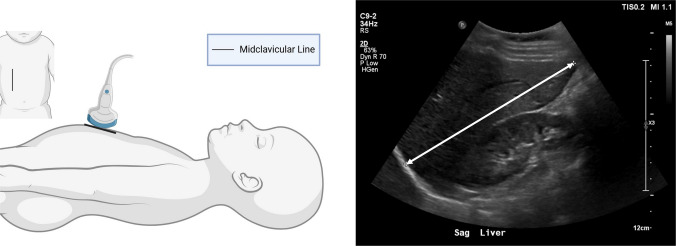
Liver ultrasound measurement in sagittal (Sag) view. Craniocaudal dimension of the right lobe, measured from the dome to the inferior tip of the liver along the midclavicular line. The ultrasound image shown is from an 11-year-old female and illustrates the measurement technique. Created in BioRender (BioRender.com, Toronto, Ontario, Canada). Wieczkowski, S. (2026) https://BioRender.com/ew94bnh

All ultrasound studies were conducted following standardized institutional protocols. Liver length was defined as the craniocaudal dimension of the right lobe along the midclavicular line, measured from the superior dome of the liver to the inferior tip (Fig. 1). Measurements were performed with the patient in the supine position. The transducer was placed longitudinally in the midclavicular line below the right costal margin. The liver dome should not be obscured by reverberation artifacts from the lung [10].

Liver size was modeled as a continuous outcome using Generalized Additive Models for Location, Scale, and Shape (GAMLSS) with application of Box–Cox Power Exponential (BCPE) distribution [11]. BCPE allows us to account for age-related changes in the average size (location), variability (spread), and asymmetry (skewness) of liver measurements. The parameter for tail heaviness (τ) was kept constant across ages. Location (μ), spread (σ), and skewness (ν) were modeled as smooth functions of age using penalized B-splines.

Model adequacy was evaluated using 3 different approaches: (1) residual versus age scatter plots with locally estimated scatterplot smoothing (LOESS) smoothing to identify systematic age-related patterns or heteroscedasticity that might indicate model misspecification across the developmental spectrum; (2) quantile–quantile plots to assess adherence to standard normal distribution; (3) histogram analysis of residual distribution to visually confirm normality assumptions.

From the fitted BCPE model age-specific reference centiles were computed at 5th, 15th, 50th, 85th, and 95th percentiles (P). The reference interval (or “normal range”) was defined as P5–P95, where P5 is the Minimum Normal (Min) and P95 the Maximum Normal (Max). P85–P95 is referred to as the upper-normal band (high-normal) and P5–P15 as the lower-normal band (low-normal); values above P95 or below P5 were considered abnormal. The decision to report P15 and P85 was made to create a gray zone, since in clinical practice, there are factors other than age or height that can affect liver size.

Data preprocessing was performed in Python (version 3.13.5; Python Software Foundation, Wilmington, DE, USA) using the pandas (version 2.2.3) [12], NumPy (version 2.1.3) [13], and re (regular expressions) (Python Software Foundation, Wilmington, DE, USA) libraries. GAMLSS were fitted in R (version 4.5.0; R Foundation for Statistical Computing, Vienna, Austria) using the gamlss package (version 5.5).

## Results

**Fig. 2:**
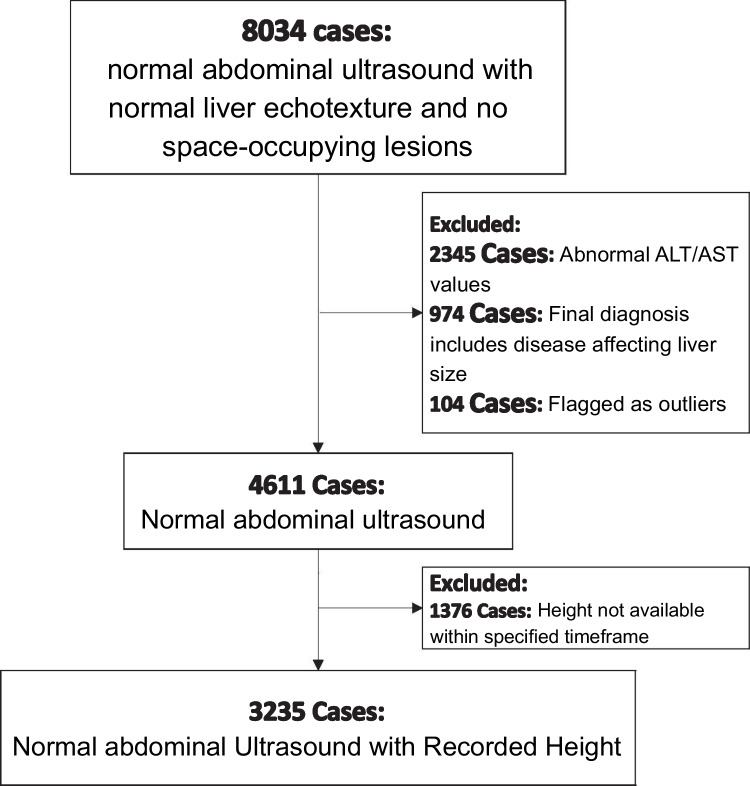
Patient selection flowchart illustrating cohort identification, inclusion and exclusion criteria, and the final study population. **ALT**: alanine aminotransferase; **AST**: aspartate aminotransferase

A total of 4611 abdominal ultrasound examinations met the inclusion criteria during the 10-year study period. The case selection process is detailed in Fig. 2. The study population consisted of 2055 males (44.6%) and 2556 females (55.4%). Further demographic characteristics are shown in Table 1.

**Table 1:** Demographic features of the included cases

Characteristics	Total (n = 4611)
Male: n (%)	2055 (44.6%)
Female: n (%)	2556 (55.4%)
Age in years: median (IQR)	5.91 (1.88–12.47)
Race: n (%)	
White	2774 (60.16%)
Black or African American	663 (14.38%)
Asian	205 (4.45%)
Native American or Alaska Native	14 (0.3%)
Native Hawaiian or Other Pacific Islander	2 (0.04%)
Other/Multiracial/Unknown*	953 (20.67%)
Ethnicity: n (%)	
Hispanic or Latino	405 (8.78%)
Not Hispanic or Latino	3710 (80.5%)
Unknown*	496 (10.7%)

*Unknown: not available or chose not to disclose

*IQR*, interquartile range

**Fig. 3:**
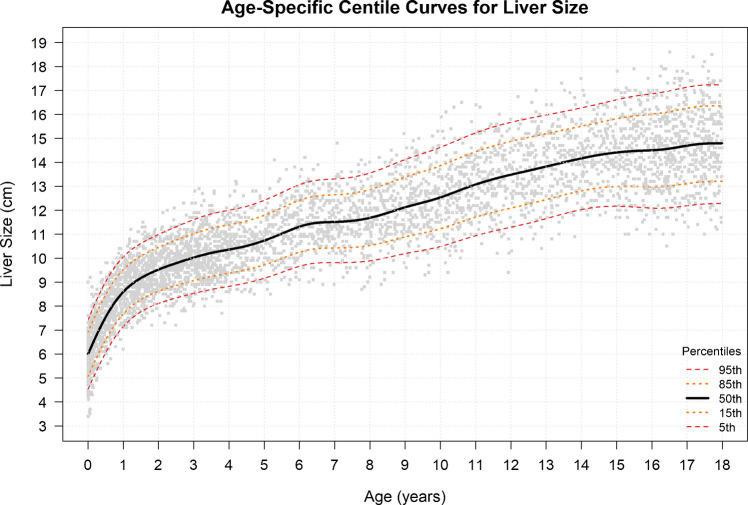
Liver measurement centile curve by age. Liver size is reported in centimeters (cm), and age is reported in years

Using the GAMLSS model, we generated minimum, maximum, and reference ranges for liver size based on age, as shown in Fig. 3, Table 2, and Online Resource 2. Reference ranges were reported according to the predefined age bins. The smallest number of patients was observed in the 9–11.99-month bin (n = 113), whereas the largest number was in the 3–3.99-year bin (n = 379). At birth, the minimum liver size was 4.9 cm, with 4.9–5.5 cm defined as the low normal range. Liver size increased progressively with age, reaching a maximum of 17.2 cm in adolescents aged 17–17.99 years, with 16.3–17.2 cm defined as the high normal range.

**Table 2:** Values of the 5th, 15th, 50th, 85th, and 95th percentiles for the different ages (in years)

Age (years)	No. of Patients	P5 (Min)	P15	P50	P85	P95 (Max)
0–0.24	322	4.9	5.5	6.4	7.3	7.9
0.25–0.49	148	5.7	6.2	7.2	8.1	8.7
0.50–0.74	141	6.4	6.9	7.9	8.8	9.3
0.75–0.99	113	6.9	7.5	8.4	9.3	9.8
1–1.49	248	7.5	8.0	8.9	9.8	10.4
1.5–1.99	220	8.0	8.5	9.4	10.3	10.8
2–2.99	378	8.3	8.9	9.8	10.8	11.3
3–3.99	379	8.7	9.2	10.2	11.2	11.8
4–4.99	194	9.0	9.5	10.5	11.6	12.2
5–5.99	180	9.4	10.0	11.0	12.1	12.7
6–6.99	187	9.8	10.4	11.5	12.6	13.3
7–7.99	168	9.8	10.4	11.6	12.7	13.4
8–8.99	172	10.0	10.7	11.9	13.1	13.8
9–9.99	194	10.3	11.0	12.3	13.6	14.4
10–10.99	185	10.7	11.5	12.8	14.2	14.9
11–11.99	157	11.1	11.9	13.3	14.7	15.5
12–12.99	153	11.5	12.3	13.7	15.0	15.8
13–13.99	155	11.9	12.6	14.0	15.4	16.1
14–14.99	162	12.1	12.9	14.3	15.7	16.4
15–15.99	220	12.1	13.0	14.5	15.9	16.8
16–16.99	278	12.1	13.0	14.6	16.1	17.0
17–17.99	257	12.3	13.2	14.8	16.3	17.2

All the liver size values are in centimeters. Age bins are presented as ranges; however, percentile estimates were calculated at the midpoint of each interval. **P:** percentile; **Min:** minimum; **Max:** maximum; **cm**: centimeter

**Fig. 4:**
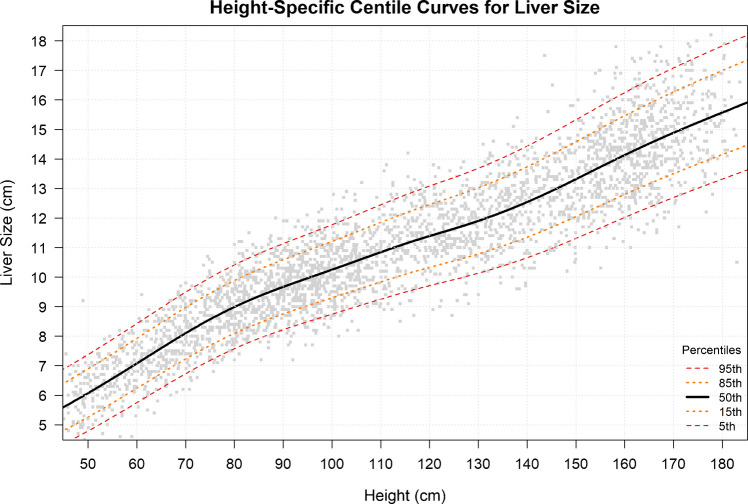
Liver measurement centile curve by height. All values are reported in centimeters (cm)

In addition to the age-based model, a subset of 3235 examinations (70%) with recorded height was used for GAMLSS modeling to generate height-based reference ranges. The model was fitted to the complete dataset (Fig. 4), but reference ranges were reported only for height bins containing at least 100 cases, to ensure adequate representation and avoid overfitting or underfitting. The resulting reference ranges are summarized in Table 3. The lowest number of cases was observed in the 170–179 cm height bin (n = 142), whereas the highest number was in the 90–99 cm bin (n = 397). Liver size increased in a more linear manner with height than with age. Reference ranges were reported up to 179 cm in height, with a maximum liver size of 17.5 cm. Values between 16.6 and 17.5 cm were considered within the high normal range.

**Table 3:** Values of the 5th, 50th, and 95th percentiles for the different heights (in centimeters)

Height (cm)	No. of Patients	P5 (Min)	P15	P50	P85	P95 (Max)
50–59	150	5.3	5.7	6.6	7.4	7.9
60–69	196	6.2	6.7	7.6	8.5	9.0
70–79	245	7.2	7.7	8.6	9.5	10.0
80–89	315	7.9	8.4	9.4	10.3	10.8
90–99	397	8.5	9.0	10.0	10.9	11.5
100–109	274	9.0	9.6	10.5	11.5	12.1
110–119	195	9.5	10.1	11.1	12.2	12.8
120–129	223	9.9	10.6	11.6	12.7	13.4
130–139	222	10.4	11.1	12.2	13.4	14.0
140–149	180	11.0	11.7	12.9	14.1	14.9
150–159	280	11.7	12.4	13.7	15.0	15.8
160–169	320	12.4	13.2	14.5	15.9	16.7
170–179	142	13.0	13.8	15.2	16.6	17.5

All the liver size values are in centimeters. Height bins are presented as ranges; however, percentile estimates were calculated at the midpoint of each interval. **P:** percentile; **Min:** minimum; **Max:** maximum; **cm**: centimeter

**Fig. 5:**
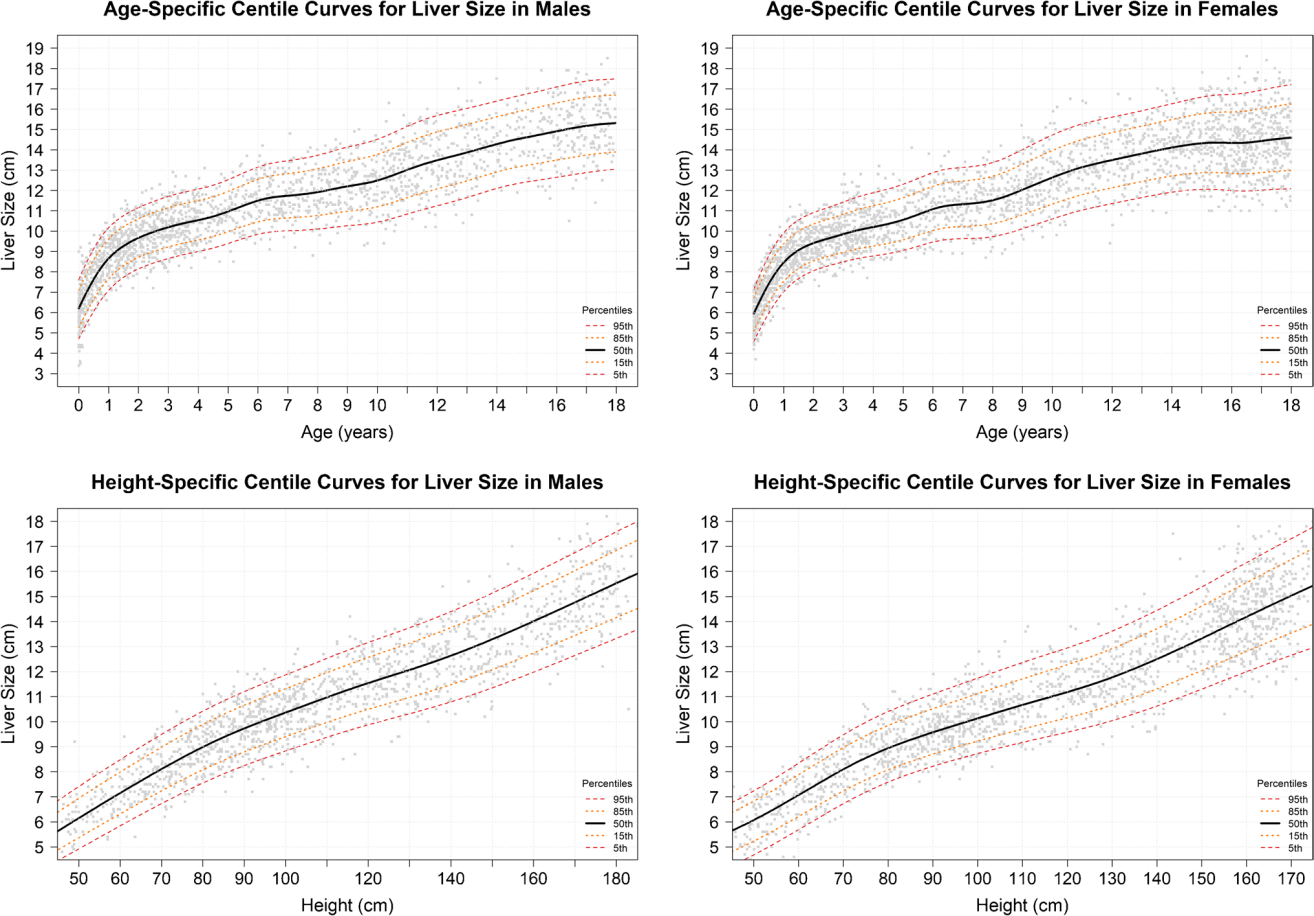
Age- and height-specific centile curves stratified by sex (male and female). Age is reported in years, and height is reported in centimeters (cm)

To characterize sex-related differences, we also created separate reference ranges for males and females (Fig. 5), which are presented in Tables 4 and 5. Sex-stratified reference ranges were reported only for height bins containing at least 50 cases.

**Table 4:** Values of the 5th, 15th, 50th, 85th, and 95th percentiles of liver size (cm) by age (years), stratified by sex

Age (years)	P5 (Min)		P15		P50		P85		P95 (Max)	
	M	F	M	F	M	F	M	F	M	F
0–0.24	5.1	4.9	5.6	5.5	6.6	6.3	7.5	7.2	0.8.0	7.6
0.25–0.49	5.8	5.6	6.3	6.2	7.3	7.1	8.3	7.9	8.8	8.4
0.50–0.74	6.4	6.3	7.0	6.8	7.9	7.7	8.9	8.6	9.5	9.1
0.75–0.99	6.9	6.8	7.5	7.3	8.4	8.2	9.4	9.2	10.0	9.7
1–1.49	7.5	7.4	8.0	7.9	9.0	8.8	10.0	9.7	10.5	10.3
1.5–1.99	8.0	7.9	8.5	8.4	9.5	9.3	10.4	10.2	11.0	10.8
2–2.99	8.4	8.3	9.0	8.8	10.0	9.6	10.9	10.6	11.5	11.2
3–3.99	8.8	8.7	9.4	9.1	10.4	10.1	11.3	11.1	11.9	11.7
4–4.99	9.2	8.9	9.7	9.4	10.7	10.4	11.7	11.4	12.3	12.1
5–5.99	9.6	9.3	10.2	9.8	11.2	10.8	12.3	11.9	12.8	12.6
6–6.99	10.0	9.6	10.6	10.2	11.7	11.3	12.7	12.4	13.4	13.1
7–7.99	10.0	9.7	10.7	10.3	11.8	11.4	12.9	12.5	13.6	13.2
8–8.99	10.2	9.9	10.9	10.6	12.1	11.7	13.2	13.0	13.9	13.7
9–9.99	10.3	10.4	11.1	11.1	12.3	12.3	13.6	13.6	14.3	14.4
10–10.99	10.6	10.8	11.4	11.6	12.7	12.9	14.1	14.3	14.8	15.0
11–11.99	11.1	11.2	11.9	12.0	13.3	13.3	14.6	14.7	15.4	15.5
12–12.99	11.4	11.5	12.3	12.3	13.7	13.7	15.1	15.0	15.9	15.8
13–13.99	11.9	11.8	12.7	12.6	14.1	14.0	15.4	15.3	16.2	16.1
14–14.99	12.3	12.0	13.1	12.8	14.5	14.2	15.8	15.6	16.6	16.4
15–15.99	12.5	12.0	13.4	12.9	14.8	14.4	16.1	15.8	16.9	16.7
16–16.99	12.8	12.0	13.6	12.8	15.1	14.4	16.5	15.9	17.3	16.8
17–17.99	13.0	12.1	13.8	12.9	15.3	14.5	16.7	16.1	17.4	17.1

Age bins are presented as ranges; however, percentile estimates were calculated at the midpoint of each interval. **P:** percentile; **Min:** minimum; **Max:** maximum; **M:** Male; **F:** Female; **cm**: centimeter

**Table 5:** Values of the 5th, 15th, 50th, 85th, and 95th percentiles of liver size (cm) by height (cm), stratified by sex

Height (cm)	P5 (Min)		P15		P50		P85		P95 (Max)	
	M	F	M	F	M	F	M	F	M	F
50–59	5.4	5.2	5.8	5.7	6.7	6.6	7.5	7.3	8.0	7.8
60–69	6.3	6.2	6.8	6.7	7.6	7.6	8.5	8.4	9.0	8.9
70–79	7.2	7.2	7.7	7.7	8.6	8.6	9.5	9.4	10.0	10.0
80–89	7.9	7.9	8.5	8.4	9.4	9.3	10.3	10.2	10.8	10.8
90–99	8.5	8.5	9.1	9.0	10.1	9.9	11.0	10.8	11.5	11.4
100–109	9.1	9.0	9.7	9.5	10.7	10.4	11.6	11.4	12.2	12.1
110–119	9.6	9.4	10.2	9.9	11.3	10.9	12.3	12.0	12.9	12.7
120–129	10.1	9.8	10.7	10.4	11.8	11.5	12.9	12.6	13.5	13.3
130–139	10.5	10.3	11.2	11.0	12.3	12.1	13.4	13.3	14.1	14.0
140–149	11.1	11.0	11.8	11.7	13.0	12.9	14.1	14.2	14.8	14.9
150–159	11.7	11.7	12.4	12.4	13.6	13.8	14.8	15.1	15.5	15.9
160–169	12.3	12.3	13.1	13.2	14.4	14.6	15.6	16.0	16.3	16.8
170–179	13.0	-	13.8	-	15.1	-	16.4	-	17.2	-

Height bins are presented as ranges; however, percentile estimates were calculated at the midpoint of each interval. **P:** percentile; **Min:** minimum; **Max:** maximum; **M:** Male; **F:** Female; **cm**: centimeter

**Fig. 6:**
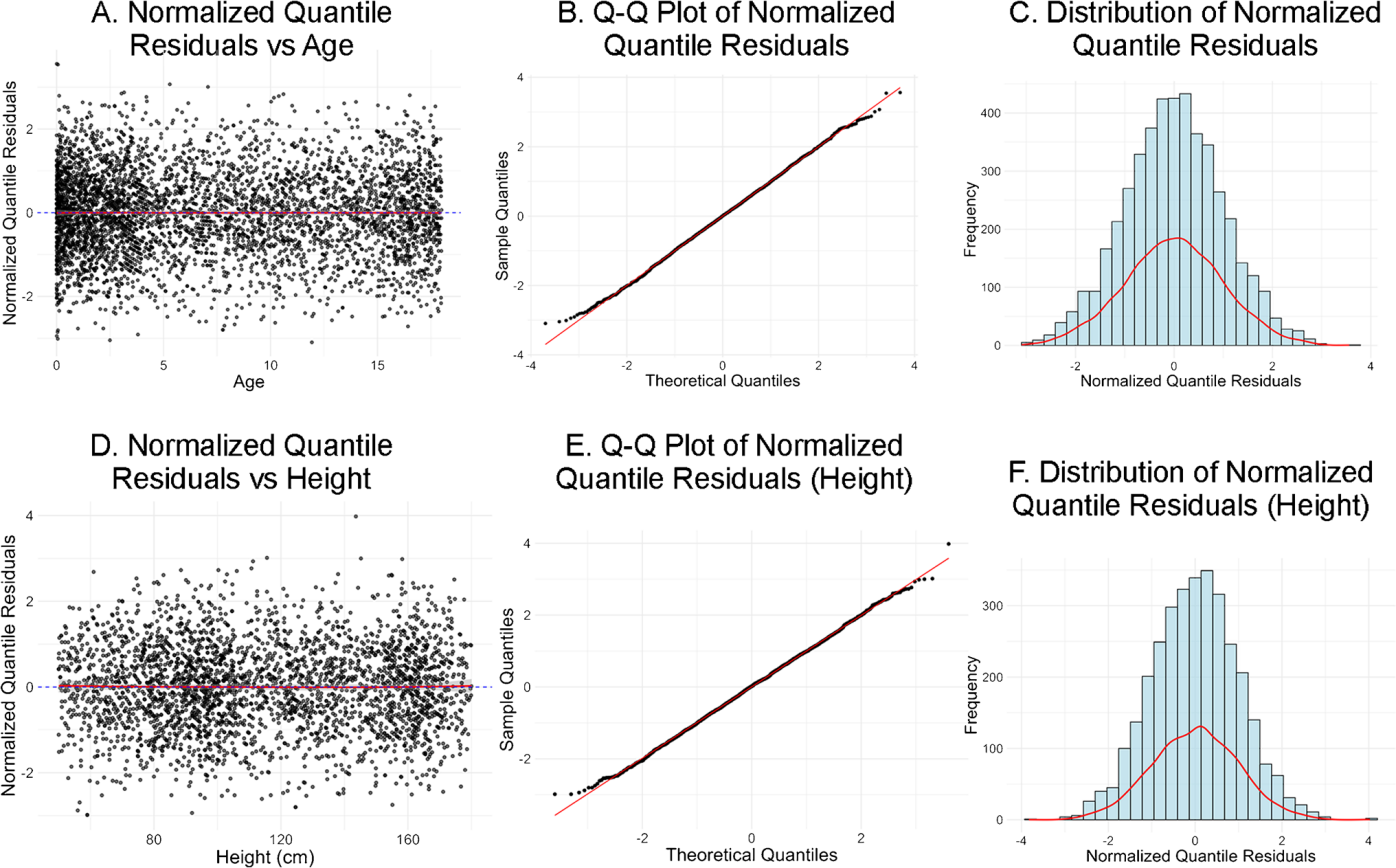
Evaluation of model fit using normalized quantile residual diagnostics. **A** shows normalized quantile residuals plotted against age, and **D** shows normalized quantile residuals plotted against height. **B** and **E** show Q–Q plots demonstrating close agreement with the theoretical normal distribution within the central quantile range of approximately –2 to + 2. **C** and **F** show histograms of the residuals, demonstrating an approximately normal, bell-shaped distribution centered a round zero. **Q–Q:** Quantile–Quantile

The normalized quantile residuals plotted against age and height were well centered around zero across the entire age range, with no discernible systematic trend, showing that the model provided an unbiased fit. The red trend line also closely followed the reference line, confirming the absence of systematic over- or underestimation at any age (Fig. 6). The Quantile–Quantile (Q–Q) plot of normalized quantile residuals demonstrates that the residuals align closely with the theoretical normal distribution within the central range of approximately –2 to + 2 quantiles, indicating a good overall model fit in the region corresponding to the 5th to 95th percentile data (Fig. 6). Finally, visual inspection of the histogram of normalized quantile residuals demonstrated an approximately normal distribution, characterized by a bell-shaped pattern centered around zero with a symmetric spread toward both tails (Fig. 6).

## Discussion

Ultrasound-based liver size nomograms for the pediatric population up to the age of 18 years are presented based on the subjects’ ages and height (Tables 1 and 2, and Figs. 3 and 4). Age is the most commonly used parameter in previous literature, as liver growth and development are expected in healthy children [1]. Additionally, age is typically the most readily available demographic information in the pediatric radiologist's daily clinical practice. Height, although not always readily accessible, has been shown to be a reliable parameter for adjusting age-height incompatibility in some patients, particularly during adolescence [7, 14, 15].

The primary motivation for this study was significant heterogeneity and limitations of previously published normal ultrasound-based liver size nomograms [1]. This limitation leads to variation in practice at our hospital, which has a large number of radiologists. The most commonly used reference is the Konuş et al. paper [16]. Some radiologists review the range of sizes reported in the systematic review by Calle-Toro et al. and select the cutoff they consider most appropriate [1]. Others diagnose hepatomegaly based on the overall appearance of hepatic bulk rather than on numeric measurements.

A key shortcoming of prior studies is the restricted age ranges covered. None of the prior publications established a normal reference range encompassing the entire pediatric population. The most comprehensive study, conducted by Konus et al., included participants from newborns up to 200 months of age (approximately 16.7 years) [16]. Two studies covered children up to 15 years, one extended to 12 years, and another only to 7 years [6, 7, 17, 18]. In contrast, the current study aimed to establish a comprehensive reference range spanning the full pediatric age range from birth to 18 years.

Another important implication of existing literature is the relatively small number of participants included in each study. Sample size is a critical determinant when establishing a normal reference range, as insufficient numbers increase the risk of overfitting and may result in a poor representation of the population. Reviewing previously published studies shows that many had small sample sizes, at least within specific age bins. For instance, in the study by Konus et al., the smallest age bin (180–200 months) included only 12 participants [16]. In the Thapa et al. study, the 145–180-month age group contained only 11 cases, while Dhingra et al. reported just 10 male participants in the 1–3-month age range [6, 17].

In contrast, the current study included substantially larger numbers across all age and height bins. The smallest age bin in this dataset comprised 113 participants (9–11.99 months), and the smallest height bin contained 142 participants (170–179 cm). In fact, in this study, reference ranges were not provided when the bin had fewer than 100 cases because of the risk of overfitting. This is why we did not report reference ranges for patients with heights less than 50 cm or greater than 180 cm. These larger sample sizes help minimize overfitting and ensure a more reliable representation of the pediatric population.

The impact of small sample sizes is also apparent in the fluctuations of normal liver size reported in previous studies. For example, Dhingra et al.reported a mean liver size of 11.9 cm for males aged 2–4 years, which unexpectedly increased to 14.7 cm for ages 4–6 years, then decreased to 12.3 cm at 6–8 years and rose again to 14.1 cm at 8–10 years [17]. This pattern implies that, paradoxically, the reported normal liver size of a 4-year-old male was greater than that of a 10-year-old male. Such inconsistencies are likely attributable to insufficient sample sizes in specific age subgroups.

Finally, when comparing results across prior studies, notable variability between published nomograms becomes evident. For example, reported maximum liver size for a 5-year-old child varied widely, ranging from 8.86 cm to 12.5 cm across different publications [16, 18]. While ethnic differences among study populations may partly account for these discrepancies, the magnitude of variation remains unusually large. This raises uncertainty for radiologists in clinical practice which reference range should be considered reliable.

Previous studies have shown that liver growth rate changes with age [19]. Eze et al. reported that liver length increases most rapidly during the first year of life [19]. Noda et al. also found that liver volume expands rapidly in infancy and continues to grow gradually until adolescence [20]. A similar pattern was observed in this study, demonstrating rate of liver growth is highest during the first year. For this reason, normal liver nomogram in the first year is divided into smaller age intervals (every 3 months) to better capture this rapid early change in normal liver size.

After the first year of life, rate of liver growth decreases but continues to increase gradually throughout childhood and adolescence. At the end of adolescence, liver growth rate slows further and approaches a near plateau. This trend is also reflected in the current study (Table 2): with median (P50) liver length increased by approximately 2 cm during the first year (from 6.4 cm to 8.4 cm), followed by a 1 cm increase in the second year (from 8.4 cm to 9.4 cm). Thereafter, liver growth rate is between 0.3 and 0.5 cm per year until around 15 years of age, after which it declines to approximately 0.1–0.2 cm per year.

While age is the most convenient and readily available variable for creating a nomogram, previous studies have shown that height is one of the key factors associated with liver size [21]. Specifically, the growth spurt can act as a confounding factor, leading to considerable variation in height among individuals within the same age group during adolescence [22]. Therefore, an additional nomogram was developed in this study based on patient height. Although patient height may not always be available, using reference ranges derived from height can provide a more accurate and individualized assessment of liver size.

Examination of the height-based nomogram demonstrates a more linear relationship between height and liver size compared to age-based model. The data can be divided into three segments based on height. Between 50 and 90 cm in height, each 10-cm increase corresponds to an approximate 0.8–1.0 cm increase in liver size. From 90 to 140 cm, the rate of increase was lower, with each 10-cm increase in height associated with a 0.5–0.6 cm increase in liver size. Beyond 140 cm, the rate of increase rose slightly, with each additional 10 cm in height corresponding to a 0.7–0.8 cm increase in liver size.

In both nomograms, the 85th (P85) and 95th (P95) percentiles are reported to define the upper range of normal values. While it is often convenient to establish a single cutoff point for biochemical markers or anthropometric measurements, physiological processes typically occur along a continuum rather than beginning abruptly at a specific threshold [23]. This concept is well recognized in other clinical parameters such as blood pressure, fasting blood glucose, and triglyceride levels, where a “gray zone” exists between normal and abnormal values. Accordingly, in both the age-based and height-based nomograms, the P85 and P95 percentiles represent a “high-normal” range. Although liver sizes between P85 and P95 may still be considered normal, interpreting these findings in conjunction with the patient’s overall clinical data is recommended.

Two separate sets of reference ranges were also created in this study for males and females to elucidate differences between them. When height-based reference ranges for male and female subjects were compared, the 50th percentiles were largely similar, with differences across height bins generally limited to 1–2 mm, showing that median liver size did not differ substantially between sexes. A similar pattern was observed for the maximum (P95) values across most height categories, except in the 160–170 cm bin, in which a 0.5-cm higher value was observed in females. This isolated difference in a single bin was considered likely to reflect overfitting rather than a true biological difference.

The proposed liver size nomograms from the current study demonstrate higher normal reference ranges compared to prior studies with similar measurement techniques. These differences may reflect variations in sample size and ethnic composition of the study populations. Waelti et al. reported normal liver dimensions in a Central European pediatric population, finding that the right lobe of the liver was approximately 1–2 cm larger in Central European children compared with non-Caucasian populations of the same age [24]. Although their study used a different measurement technique, which limits direct comparison, the findings are consistent with those reported here. Similarly, comparison with the study by Konus et al., which employed the same technique and included subjects ranging in age from infancy to nearly 17 years, demonstrates a comparable pattern [16]. In the early childhood, the difference between the current data and theirs was approximately 1 cm, increasing with age to exceed 2 cm in older children [16].

The higher upper limits observed in the current study compared with previously published pediatric data prompted comparison with normal liver size in the adult population, specifically the final age bin (17–18 years). In adults, a liver length exceeding 16 cm in the midclavicular line is generally considered enlarged [25]. In the 17–18-year-old cohort, the 85th percentile (lower bound of the high-normal range) was 0.3 cm larger than this conventional cutoff, and the 95th percentile (upper bound of the high-normal range and maximum normal liver size) was 1.2 cm larger. However, a systematic review of liver measurement studies in adults found that none of the included studies demonstrated adequate rigor regarding sample size justification, measurement validity, reliability testing, or statistical methodology, and concluded that the conventional 16-cm cutoff may lack sufficient accuracy for defining hepatomegaly [25].

Moreover, another study involving 2080 adults reported that 11.5% of subjects had a liver length of more than 16 cm [21]. These findings suggest that the widely accepted 16-cm cutoff for adults lacks strong scientific and statistical justification, underscoring the need for contemporary, well-designed studies to refine the definition of normal liver size in adults.

A question that may arise from these reference ranges is how to interpret measurements that are classified as normal when assessed by age but abnormal when assessed by height, or vice versa. Previous studies have demonstrated that liver size correlates more strongly with height than with age; therefore, in such situations, using height-based centile curves is recommended.

While this study aimed to establish reference ranges that are broadly generalizable, applying these ranges to specific groups or populations should be done with caution. Anatomical variations, such as Riedel’s lobe and the beaver tail liver can alter lobar proportions, thereby influencing measured liver dimensions without indicating underlying pathology [26–28]. As a result, it is recommended that radiologists, prior to applying these standards, assess liver morphology and confirm that the evaluated liver does not represent a normal anatomic variant, since the presented reference ranges were derived from normally shaped livers rather than from anatomic variants. Qualitative features such as liver bulk may be assessed by the radiologist to determine hepatomegaly in these cases.

Another consideration is prematurity in neonates. Information on gestational age was not collected in the current data set, and this factor may particularly affect liver size during the early years of life. Further studies focusing on early childhood are necessary to evaluate the impact of prematurity on liver dimensions and to distinguish between term and preterm infants.

This study has both strengths and limitations. A retrospective design was used, which may appear less rigorous than a prospective approach; however, each design involves inherent compromises. Prospective studies maximize control but are often limited by smaller sample sizes, which may have contributed to previously mentioned questions regarding population representativeness and increased risk of model overfitting. Retrospective data, by contrast, allow analysis of thousands of data points, reducing this risk. Additionally, in routine clinical practice, liver measurements are inherently variable. They are obtained using different devices and by individual sonographers with variable techniques. This study more accurately reflects this real-world variability, whereas studies employing a single device and sonographer may provide highly controlled but less generalizable results. Consequently, prospective approaches generate efficacy-style reference ranges emphasizing control, while this retrospective approach maximizes representativeness, providing effectiveness-style reference ranges. Both approaches are complementary, and future prospective studies could be used to further refine liver reference ranges in a controlled setting, aiding radiologists in the assessment of specific conditions.

Another limitation is that the patients included in this study were identified based on an impression of a normal abdominal ultrasound. However, these assessments may have been based on previously published reference ranges, which could have led to an underestimation of normal liver size. Despite this potential limitation,in this study, most of the ranges were higher than those reported in prior studies.

Finally, it should be noted that liver size is influenced by factors beyond age and height alone. Obesity and geographic differences may affect liver dimensions. These variables were not accounted for in this analysis. Future studies incorporating body mass index or body surface area, as well as multi-regional cohorts, may further refine reference standards.

## Conclusion

Assessing liver size using ultrasound is a common approach in the evaluation and management of a wide range of liver diseases in children. This study presents an updated normal reference range for liver size in the pediatric population with nomograms based on both age and height. These reference ranges suggest that normal liver size limits are slightly higher than those reported in current literature. The large sample size, together with rigorous statistical analysis, enhances accuracy and supports their applicability in pediatric radiology clinical practice.

## Supplementary Information

Below is the link to the electronic supplementary material.

Supplementary file1 (DOCX 15.7 KB)

Supplementary file2 (DOCX 214 KB)

## Data Availability

All authors ensured that the data and materials presented complied with standards. The datasets generated during and/or analyzed during the current study are available from the corresponding author on reasonable request.
