# Supplementary material for: Sonographic pediatric liver size standards based on generalized additive modeling in a diverse population
Source: Pediatr Radiol. 2026 Apr 24;56(6):1368–79. doi: 10.1007/s00247-026-06612-3 (PMC13212396; doi:10.1007/s00247-026-06612-3)
Supplement: Supplementary file 1 — Supplementary file1 (DOCX 15.7 KB) [file 247_2026_6612_MOESM1_ESM.docx]

**Online Resource 1:** List of Excluded Diagnoses and Corresponding ICD-10 Codes for Patients Initially Identified.

| ICD-10 Codes | Diseases/Conditions |
| --- | --- |
| K71.* | Toxic liver disease |
| K72.* | Hepatic failure, not elsewhere classified |
| K73.* | Chronic hepatitis, not elsewhere classified |
| K74.* | Fibrosis and cirrhosis of liver |
| K75.* | Other inflammatory liver diseases |
| K76.* | Other diseases of liver |
| K77.* | Liver disorders in diseases classified elsewhere |
| B15.* | Acute hepatitis A |
| B16.* | Acute hepatitis B |
| B17.* | Other acute viral hepatitis |
| B18.* | Chronic viral hepatitis |
| B19.* | Unspecified viral hepatitis |
| C22.* | Malignant neoplasm of liver and intrahepatic bile ducts |
| K83.* | Other diseases of biliary tract |
| I85.* | Esophageal varices |
| I82.0 | Budd-Chiari syndrome |
| Q44.* | Congenital malformations of gallbladder, bile ducts and liver |
| D18.03 | Hemangioma of intra-abdominal structures |
| I50 | Heart failure |

* indicates all subcodes within the ICD-10 category.
