# Supplementary figures and images for: Sonographic pediatric liver size standards based on generalized additive modeling in a diverse population
Source: Pediatr Radiol. 2026 Apr 24;56(6):1368–79. doi: 10.1007/s00247-026-06612-3 (PMC13212396; doi:10.1007/s00247-026-06612-3)

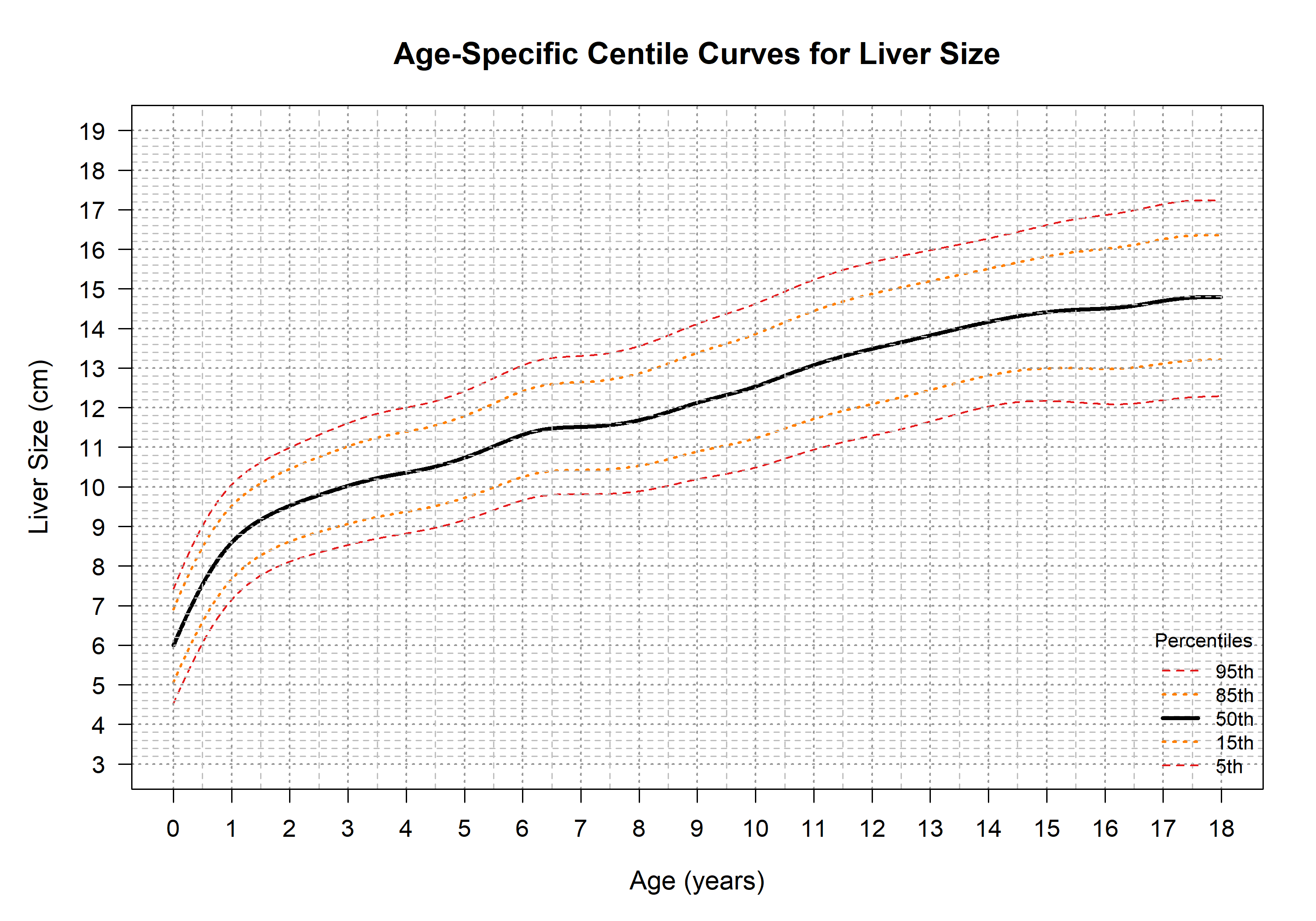


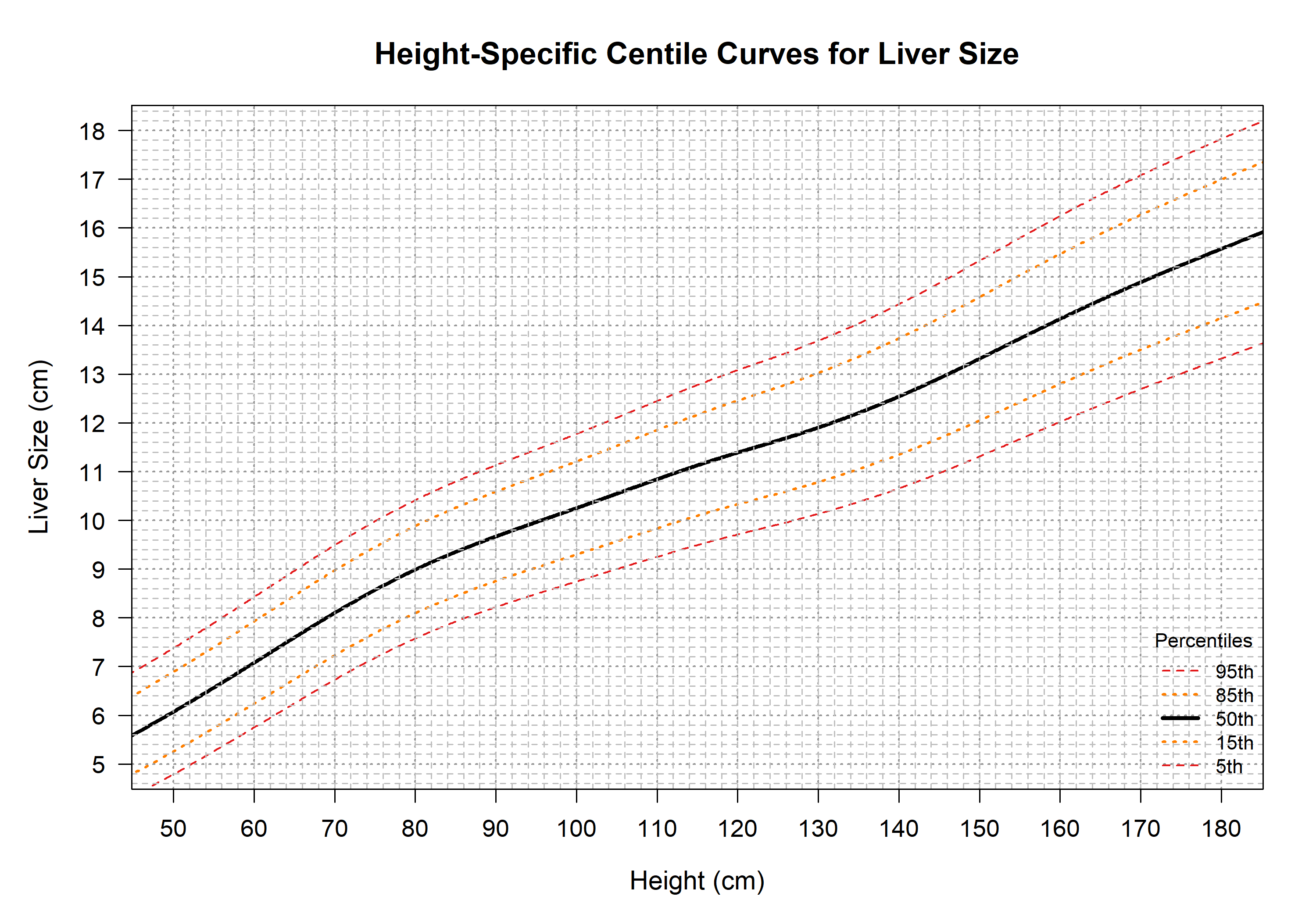

Supplement: Supplementary file 2 — Supplementary file2 (DOCX 214 KB) [file 247_2026_6612_MOESM2_ESM.docx]
